# Association of the tumor necrosis factor-alpha promoter polymorphism with change in triacylglycerol response to sequential meals

**DOI:** 10.1186/s12937-016-0190-9

**Published:** 2016-07-25

**Authors:** Kim G. Jackson, Yue Li, Miriam F. Ryan, Eileen R. Gibney, Lorraine Brennan, Helen M. Roche, Christine M. Williams, Julie A. Lovegrove, Karani S. Vimaleswaran

**Affiliations:** 1 Hugh Sinclair Unit of Human Nutrition, Department of Food and Nutritional Sciences, University of Reading, Reading, UK; 2 Institute for Cardiovascular and Metabolic Research (ICMR), University of Reading, Reading, UK; 3 UCD Institute of Food & Health, University College Dublin, Dublin, Ireland

**Keywords:** Postprandial lipaemia, Triacylglycerol, TNFA promoter polymorphism, BMI, MECHE

## Abstract

**Background:**

Reported associations between Tumor Necrosis Factor-alpha (TNFA) and the postprandial triacylglycerol (TAG) response have been inconsistent, which could be due to variations in the *TNFA* gene, meal fat composition or participant’s body weight. Hence, we investigated the association of *TNFA* polymorphism (−308G → A) with body mass index (BMI) and postprandial lipaemia and also determined the impact of BMI on the association of the polymorphism with postprandial lipaemia.

**Methods:**

The study participants (*n* = 230) underwent a sequential meal postprandial study. Blood samples were taken at regular intervals after a test breakfast (*t* = 0, 49 g fat) and lunch (*t* =330 min, 29 g fat) to measure fasting and postprandial lipids, glucose and insulin. The Metabolic Challenge Study (MECHE) comprising 67 Irish participants who underwent a 54 g fat oral lipid tolerance test was used as a replication cohort. The impact of genotype on postprandial responses was determined using general linear model with adjustment for potential confounders.

**Results:**

The -308G → A polymorphism showed a significant association with BMI (*P* = 0.03) and fasting glucose (*P* = 0.006), where the polymorphism explained 13 % of the variation in the fasting glucose. A 30 % higher incremental area under the curve (IAUC) was observed for the postprandial TAG response in the GG homozygotes than A-allele carriers (*P* = 0.004) and the genotype explained 19 % of the variation in the IAUC. There was a non-significant trend in the impact of BMI on the association of the genotype with TAG IAUC (*P* = 0.09). These results were not statistically significant in the MECHE cohort, which could be due to the differences in the sample size, meal composition, baseline lipid profile, allelic diversity and postprandial characterisation of participants across the two cohorts.

**Conclusions:**

Our findings suggest that *TNFA* -308G → A polymorphism may be an important candidate for BMI, fasting glucose and postprandial TAG response. Further studies are required to investigate the mechanistic effects of the polymorphism on glucose and TAG metabolism, and determine whether BMI is an important variable which should be considered in the design of future studies.

**Trial registration:**

NCT01172951.

## Background

The magnitude and duration of the postprandial triacylglycerol (TAG) response have been shown to be highly variable between individuals due to genetic and dietary factors [1]. Although multiple candidate genes in lipoprotein metabolism pathways have been identified [2–4], studies have indicated that genetic variants in tumor necrosis factor-alpha (TNF-α), a cytokine released from macrophages during inflammation, may also play an important role in modulating the lipid response to high-fat meal ingestion [5, 6]. This is based on evidence, where change in serum TAG was linked to change in TNF-α after high-fat meal consumption [7]. Under post absorptive conditions, the infusion of a relatively low dose of TNF-α into healthy participants stimulated lipolysis and altered liver fat metabolism [8]. These studies suggest that *TNFA* could be an important candidate gene for postprandial TAG metabolism.

The most widely studied single nucleotide polymorphism (SNP) [−308 G → A (rs1800629)] in the promoter of *TNFA* gene has been shown to alter its transcriptional regulation and production [9]. Two studies have shown an association between the *TNFA* -308G → A polymorphism and postprandial TAG [10, 11], while two studies failed to show an effect [6, 12]. Furthermore, TNF-α is expressed in adipocytes and correlates with the degree of adiposity [13]. The effect of this SNP on lipid and glucose metabolism has been proposed to be dependent on body mass index (BMI) [14] and differences in BMI could be a reason for the discrepancy in the study findings. Hence, in the present data analysis, we examined the association of the *TNFA* -308G → A SNP with BMI, fasting and postprandial lipid, non-esterified fatty acid (NEFA), glucose and insulin after consumption of sequential meals in 230 participants (England) genotyped for the *TNFA* polymorphism. In addition, we also examined the impact of BMI on the association of the *TNFA* polymorphism with fasting and postprandial lipid, glucose and insulin response to sequential meal ingestion. To confirm our findings, we used an Irish postprandial study, Metabolic Challenge Study (MECHE) [15], as a replication cohort.

## Methods

### Participants

The data analysis was performed using postprandial responses from 230 healthy participants (mean age of 52 (range 22–71) y and BMI 26.3 (range 19.6–31.9) kg/m^2^) who had undergone the same sequential test meal protocol at the University of Reading, England, between 1997 and 2007, as previously described [16, 17]. The studies were reviewed by the University of Reading Research Ethics Committee and the West Berkshire Health Authority Ethics Committees, and each volunteer gave written informed consent before participating. All protocols and procedures were performed according to the Declaration of Helsinki.

### Postprandial meal protocol

The participants underwent a novel sequential meal protocol, as previously described [18]. Briefly, participants were asked to refrain from alcohol and strenuous exercise on the day before the study visit and were provided with a low fat (<10 g fat) evening meal. After a 12 h overnight fasting, participants were cannulated and a blood sample was taken to measure fasting levels of lipids, glucose, NEFA and insulin. Following a test breakfast (0 min) and lunch (330 min), blood samples were taken at 60 min intervals until 480 min after the breakfast meal. The nutritional composition of the breakfast was 3.9 MJ energy, 111 g carbohydrate, 19 g protein and 49 g fat and the lunch was 2.3 MJ energy, 63 g carbohydrate, 15 g protein and 29 g fat. The type of fat contained within the test meals was predominately saturated, with 29 g of saturated fatty acids (SFA) in the breakfast and 14 g of SFA in the lunch. No other food or drink except water and decaffeinated sugar free drinks was allowed during the study day.

### Measurement of Clinical and biochemical parameters

The plasma lipid profile, glucose and insulin were measured as previously described [17]. All samples for each individual were analysed within a single batch and the inter-assay coefficient of variation for the assays were less than 5 %. The homeostasis model assessment of insulin resistance (HOMA-IR) was calculated using the fasting glucose and insulin data [fasting insulin (pmol/l) x fasting glucose (mmol/l)]/135] [19].

### Genotyping

DNA was isolated from the buffy coat layer of 10 ml of EDTA blood using the Qiagen DNA Blood Mini Kit (Qiagen Ltd, Crawley, UK). The *TNFA* -308G → A (rs1800629) polymorphism was genotyped using a TaqMan SNP genotyping assay (Life Technologies). The genotype distribution was in Hardy-Weinberg equilibrium (*P* = 0.46).

### Replication cohort

#### Study participants

Sixty-seven participants were chosen from the Metabolic Challenge Study (MECHE) that recruited healthy volunteers (*N* = 214, age 18–60)) at University College Dublin (UCD), Ireland [15]. Ethical approval was obtained from the Research Ethics Committees at UCD and St Vincent’s University Hospital Dublin. All participants provided written informed consent. MECHE was registered under NCT01172951 at Clinicaltrials.gov.

#### Postprandial meal protocol

The participants (44 men/23 women) underwent an oral lipid tolerance test (OLTT) following a 12 h overnight fast [20]. The OLTT consisted of 100 mL Calogen (Nutricia, Ireland) combined with 50 mL Liquid Duocal (SHS Nutrition, Netherlands) which contained 54 g fat (7 g SFA, 31 g monounsaturated fatty acids, 16 g polyunsaturated fatty acids) and 16 g carbohydrate (550 kcal).

#### Measurement of clinical and biochemical parameters

Clinical chemistry analysis was performed using a RxDaytona™ chemical analyser autoanalyser (Randox Laboratories, Crumlin, UK) and Randox reagents. The plasma lipid profile, glucose and insulin were measured as previously described [15].

#### Genotyping

DNA was isolated from the buffy coat layer of EDTA blood using a Gentra Autopure LS robotic workstation (Qiagen Ltd, Crawley, UK). The *TNFA* -308G → A (rs1800629) polymorphism was genotyped by LGC Genomics (www.lgcgenomics.com) using their proprietary KASPar polymerase chain reaction technique. Genotype distribution was in Hardy-Weinberg equilibrium (*P* < 0.05).

### Statistical analysis

All statistical analyses were performed using SPSS software, version 21. All biochemical outcomes were expressed as means and SEM, checked for normality and transformed where necessary. BMI cut-off <25 kg/m^2^ (normal weight) and ≥25 kg/m^2^ (overweight/obese) was based on criteria from the World Health Organization [21]. The total area under the variable versus time curves (AUC) was calculated using the trapezium rule for postprandial TAG, glucose and insulin for 0–480 min. Incremental AUC (IAUC, 0–480 min) was calculated as AUC minus the fasting concentration. NEFA AUC and IAUC responses were calculated from the time of suppression until the end of the postprandial period (120–480 min). The impact of genotype on postprandial (AUC and IAUC) responses was determined using general linear model with adjustment for age, gender and BMI. The *χ* ^2^ test was used to compare the proportions of genotypes or alleles. Due to the small number of homozygotes (AA) in the postprandial cohorts, individuals homozygous (AA) and heterozygous (GA) for the polymorphism were grouped together for statistical analyses. *P* ≤ 0.05 was considered statistically significant.

## Results

### Association of the TNFA -308G → A polymorphism with BMI and baseline biochemical parameters

There were significant associations of the *TNFA -*308G → A polymorphism with BMI (*P* = 0.03) and fasting glucose concentration (*P* = 0.006). There was also a significant difference between the normal weight and overweight/obese groups with respect to the *TNFA -*308G → A polymorphism (*P* = 0.001), where the proportion of overweight/obese individuals with GG genotypes (79 %) was higher than that of the normal weight individuals with GG genotypes (60 %). Given the significant difference between the two BMI groups, sub-group analysis was performed in the normal weight (*n* = 94) and overweight/obese (*N* = 136) participants. The genetic effect on fasting glucose was significant only in overweight/obese group (*P* = 0.005), with 7.4 % higher concentration in GG genotype than A-allele carriers. The variation in fasting glucose that can be explained by this SNP was 13 % in the overweight/obese group. The baseline demographic and biochemical characteristics of the participants stratified by genotypes in normal weight (BMI < 25 kg/m^2^) and overweight/obese (BMI ≥ 25 kg/m^2^) participants are presented in Table 1.

**Table 1:** Fasting and postprandial metabolites and characteristics according to the *TNFA*-380G → A polymorphism stratified by body mass index in the UK postprandial cohort

	Normal weight (BMI < 25 kg/m^2^)			Overweight/obese (BMI ≥ 25 kg/m^2^)			*P* ^*^
	GG (*N* = 56)	GA + AA (*N* = 38)	*P* ^a^	GG (*N* = 108)	GA + AA (*N* = 28)	*P* ^a^	
Age (yrs)	51 ± 2	48 ± 2	0.16	54 ± 1	54 ± 2	0.96	0.09
Gender (men/women)	24/32	10/28	0.10	70/38	18/10	0.96	0.03
BMI (kg/m^2^)^b^	23.10 ± 0.20	23.20 ± 0.20	0.21	28.50 ± 0.20	28.00 ± 0.30	0.29	0.03
*Fasting metabolites* ^a^							
TC (mmol/l)	5.46 + 0.13	5.30 ± 0.15	0.97	5.81 ± 0.10	5.85 ± 0.21	0.89	0.97
TAG (mmol/l)	1.27 ± 0.70	1.11 ± 0.07	0.43	1.81 ± 0.09	1.65 ± 0.11	0.36	0.23
HDL-c (mmol/l)	1.48 ± 0.05	1.56 ± 0.07	0.73	1.24 ± 0.04	1.31 ± 0.06	0.37	0.54
LDL-c (mmol/l)	3.39 ± 0.13	3.27 ± 0.15	0.79	3.76 ± 0.09	3.79 ± 0.20	0.89	0.81
Glucose (mmol/l)	4.98 ± 0.07	4.78 ± 0.06	0.31	5.37 ± 0.07	4.99 ± 0.09	0.007	0.006
Insulin (pmol/l)^c^	36.50 ± 3.70	32.00 ± 4.30	0.51	57.60 ± 3.80	49.20 ± 6.60	0.75	0.54
NEFA (μmol/l)	525 ± 30	493 ± 26	0.39	526 ± 17	494 ± 39	0.59	0.39
HOMA-IR^c^	1.40 ± 0.20	1.20 ± 0.20	0.43	2.40 ± 0.20	2.00 ± 0.30	0.62	0.44
*Postprandial measures* ^a^							
TAG (mmol/l × 480 min)							
AUC	885 ± 51	754 ± 44	0.42	1293 ± 55	1189 ± 78	0.31	0.15
IAUC^d^	284 ± 26	191 ± 21	0.09	396 ± 24	333 ± 29	0.12	0.02
NEFA (mmol/l × 300 min)^e^							
AUC	147 ± 8	140 ± 7	0.35	157 ± 5	163 ± 7	0.29	0.92
IAUC	92 ± 6	70 ± 16	0.06	102 ± 3	110 ± 6	0.22	0.49
Glucose (mmol/l × 480 min)^f^							
AUC	2943 ± 45	2740 ± 166	0.46	3176 ± 56	2968 ± 153	0.15	0.07
IAUC	564 ± 45	514 ± 55	0.82	590 ± 32	603 ± 60	1.00	0.93
Insulin (nmol/l × 480 min)^g^							
AUC	99 ± 11	117 ± 9	0.37	141 ± 19	149 ± 30	0.28	0.33
IAUC	81 ± 9	99 ± 9	0.41	129 ± 17	122 ± 28	0.47	0.36

Values represent mean ± S.E. mean

As the AA homozygotes were rare (1.7 %), all analyses were conducted by comparing GG homozygotes A allele carriers (GA + AA)

Abbreviations: *AUC*, area under the curve, *BMI* body mass index, *HDL-c* high density lipoprotein cholesterol, *HOMA-IR* homeostasis model assessment of insulin resistance, *IAUC* incremental area under the curve, *LDL-c* low density lipoprotein cholesterol, *NEFA* non-esterified fatty acids, *TAG* triacylglycerol, *TC* total cholesterol

^a^Adjusted for age, gender, and body mass index

^b^Adjusted for age, and gender

^c^BMI < 25, GG (*N* = 34), A (*N* = 19); BMI ≥ 25, GG (*N* = 79), A (*N* = 19)

^d^BMI < 25, GG (*N* = 56), A (*N* = 37); BMI ≥ 25, GG (*N* = 106), A (*N* = 26)

^e^BMI < 25, GG (*N* = 36), A (*N* = 19); BMI ≥ 25, GG (*N* = 84), A (*N* = 21)

^f^BMI < 25, GG (*N* = 36), A (*N* = 20); BMI ≥ 25, GG (*N* = 86), A (*N* = 22)

^g^BMI < 25, GG (*N* = 15), A (*N* = 7); BMI ≥ 25, GG (*N* = 35), A (*N* = 10)

^*^ *P* values for the difference in the clinical and biochemical parameters between the genotypes in the whole group

#### Association of the TNFA -308G → A polymorphism with postprandial biochemical parameters

**Fig. 1:**
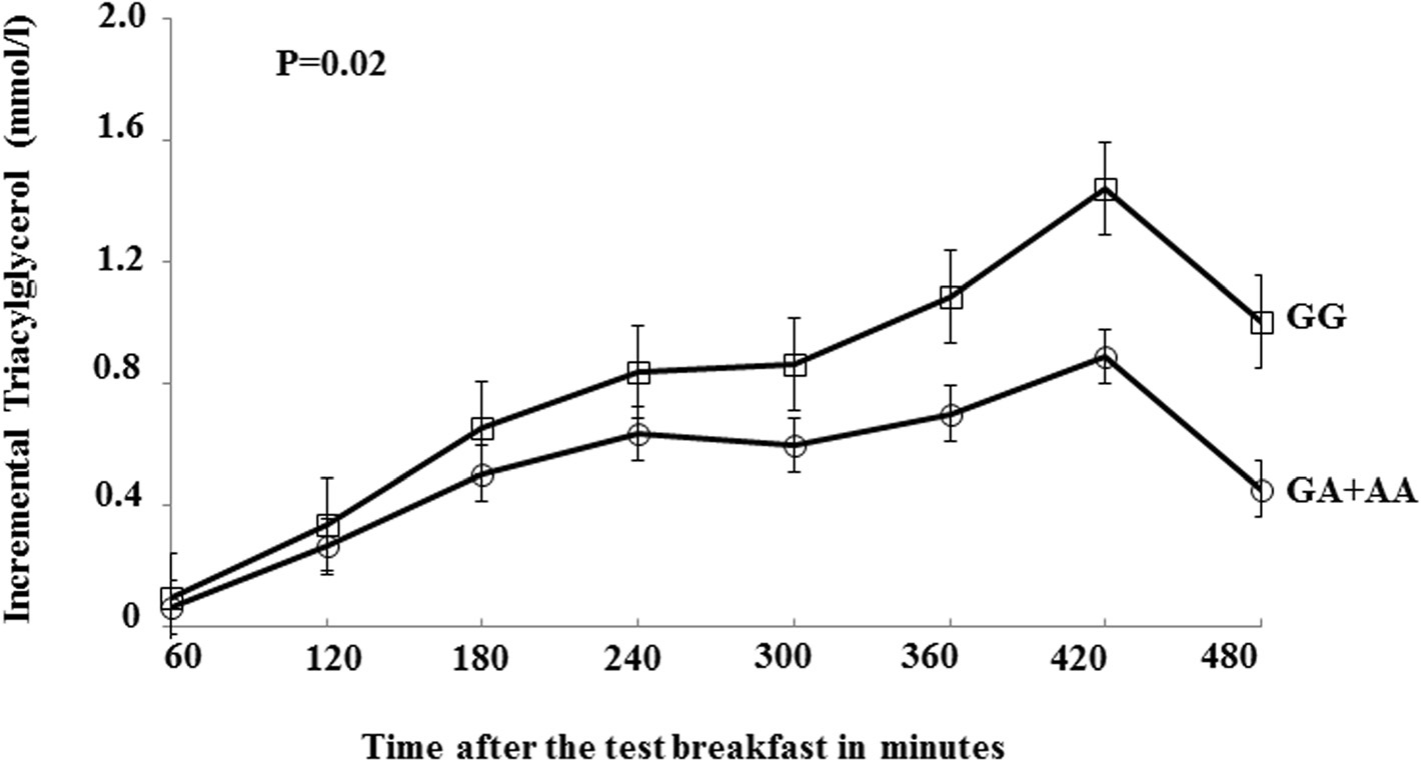
Incremental triacylglycerol response (mmol/l) according to the *TNFA −*308 G/A polymorphism. Mean (SEM) for the incremental triacylglycerol response (mmol/l) according to the *TNFA −*308 G/A polymorphism [GG genotype (*N* = 162), open squares and GA + AA genotype combination (*N* = 64, open circles)] after consumption of a test breakfast (49 g fat) at 0 min and a test lunch (29 g fat) at 330 min. *P* value represents the difference in the incremental triacylglycerol response between the genotypes (GG vs. GA + AA) of the *TNFA −*308 G/A polymorphism

Following the high-fat meals (breakfast and lunch), a significant effect of this SNP was observed on the TAG IAUC (*P* = 0.02) but not AUC (*P* = 0.15), where the GG genotype group had 30 % higher TAG IAUC than A-allele carriers (Fig. 1). The polymorphism explained 19 % of the variation in the TAG IAUC. There was no difference in postprandial NEFA, glucose or insulin responses observed between genotypes (Table 1). When stratified as BMI <25 kg/m^2^ (normal weight) and ≥25 kg/m^2^ (overweight/obese), there was a trend in the association of the SNP with TAG IAUC in the normal weight group but the findings were not statistically significant (*P* = 0.09). Given the previous gender-specific associations observed in this postprandial study [18, 22], stratification by gender was also performed and found that the genetic associations on TAG IAUC were significant in men (*GG*, 439 ± 26 mmol/l x 480 min, *n* = 92; *GA + AA*, 320 ± 30 mmol/l x 480 min, *n* = 27; *P* = 0.03) but not in women (*GG*, 249 ± 19 mmol/l x 480 min, *n* = 70; *GA + AA*, 194 ± 21 mmol/l x 480 min, *n* = 37; *P* = 0.39).

#### Replication of the findings in the Irish cohort

There was no association of the *TNFA* -308G → A polymorphism with BMI (*P* = 0.58), fasting glucose concentration (*P* = 0.46) and TAG IAUC (*P* = 0.72) (Table 2). We also did not find any effect of BMI on the association of the SNP with TAG IAUC (*P* = 0.54). Even though the findings were not statistically significant in the Irish cohort, in contrast to the UK cohort, the A allele carriers (34 %) had a tendency for higher fasting glucose and TAG IAUC, compared to GG homozygotes (66 %) (Table 2).

**Table 2:** Fasting and postprandial metabolites and characteristics according to the *TNFA*-380G → A polymorphism stratified by body mass index in the Metabolic Challenge Study (Replication cohort)

	Normal weight (BMI <25 kg/m^2^)			Overweight/obese (BMI ≥ 25 kg/m^2^)			*P* ^*^
	GG (*N* = 30)	GA + AA (*N* = 15)	*P* ^a^	GG (*N* = 14)	GA + AA (*N* = 8)	*P* ^a^	
Age	31 ± 2	27 ± 3	0.34	31 ± 2	35 ± 4	0.29	0.74
Gender (men/women)	8/22	8/7	0.08	10/4	4/4	0.32	0.38
BMI (kg/m^2^)^b^	22.70 ± 0.30	22.40 ± 0.50	0.52	26.80 ± 0.60	26.70 ± 0.50	0.85	0.58
*Fasting metabolites*							
TC (mmol/l)	4.50 ± 0.20	4.30 ± 0.20	0.88	4.30 ± 0.20	5.10 ± 0.30	0.04	0.48
TAG (mmol/l)	0.94 ± 0.07	0.86 ± 0.05	0.48	1.18 ± 0.2	1.30 ± 0.20	0.72	0.97
Glucose (mmol/l)	5.01 ± 0.08	5.20 ± 0.10	0.26	5.30 ± 0.10	5.30 ± 0.08	0.93	0.46
HDL-c (mmol/l)	1.50 ± 0.07	1.40 ± 0.09	0.95	1.20 ± 0.08	1.40 ± 0.20	0.33	0.79
Insulin (pmol/l)	34.80 ± 0.60	36.10 ± 4.90	0.71	43.80 ± 5.60	50.70 ± 6.30	0.49	0.23
NEFA (μmol/l)	590 ± 50	590 ± 90	0.73	560 ± 29	550 ± 25	0.69	0.99
HOMA-IR	2.00 ± 0.20	2.10 ± 0.30	0.69	2.50 ± 0.40	2.80 ± 0.30	0.59	0.27
TNFA (pg/ml)	10.20 ± 4.50	4.30 ± 1.80	0.59	4.10 ± 0.40	4.70 ± 0.70	0.35	0.45
*Postprandial TAG (mmol/l × 300 min)*							
AUC	176 ± 31	170 ± 41	0.74	184 ± 50	192 ± 66	0.78	0.99
IAUC	81 ± 15	84 ± 23	0.87	63 ± 26	81 ± 28	0.54	0.72
*Postprandial NEFA (mmol/l × 180 min)*							
AUC	39 ± 7	36 ± 8	0.73	26 ± 7	32 ± 10	0.58	0.96
IAUC	14 ± 3	12 ± 4	0.44	6 ± 2	3 ± 5	0.18	0.65
*Postprandial Glucose (mmol/l × 300 min)*							
AUC	483 ± 75	485 ± 108	0.89	468 ± 108	442 ± 713	0.95	0.98
IAUC	−23 ± 9	−31 ± 12	0.47	−72 ± 36	−9 ± 6	0.21	0.34
*Postprandial Insulin (pmol/l × 300 min)*							
AUC	4209 ± 806	4223 ± 1035	0.71	6257 ± 2368	5382 ± 1792	0.73	0.82
IAUC	986 ± 340	313 ± 465	0.11	2417 ± 1695	1063 ± 597	0.46	0.28

Values represent mean ± S.E. mean

As there was only one participant with AA homozygous genotype, all analyses were conducted by comparing GG homozygotes and A allele carriers (GA + AA)

Abbreviations: *AUC* area under the curve, *IAUC*, incremental area under the curve, *BMI* body mass index, *TAG* triacylglycerol, *TC* total cholesterol, *HDL-c* high density lipoprotein cholesterol

^a^Adjusted for age, gender, and BMI, wherever appropriate

^b^Adjusted for age, and gender

^*^ *P* values for the difference in the clinical and biochemical parameters between the genotypes in the whole group

## Discussion

There are two important findings in this study. Firstly, there is an independent association of *TNFA* -308G → A polymorphism with BMI and fasting glucose concentration. Secondly, there was a genotype effect observed on the TAG IAUC, with a higher response in the GG than A allele carriers after the sequential meals.

Several polymorphisms have been identified in the promoter region of the *TNFA* gene [23, 24]; however, to date, −308G → A polymorphism (rs1800629) has been the best characterized genetic variant in relation to metabolic and cardiovascular disease outcomes [24–26]. The *TNFA* -308G → A polymorphism has been linked with BMI and obesity [13, 27], where A allele carriers have been shown to have higher BMI [27]. In contrast but in line with a few studies [11, 28], we observed a higher proportion of GG homozygotes in the overweight/obese than normal weight group, where they also exhibited higher fasting glucose concentrations than the A allele carriers. Our finding is biologically plausible given that obese patients have been shown to have an abnormal postprandial lipaemia response due to postprandial changes in endogenous lipoproteins as a result of insulin resistance [29]. Hence, it is possible that GG homozygotes with higher BMI and fasting glucose concentrations are likely to exhibit higher postprandial TAG levels. BMI may therefore be an important variable that determines the impact of this polymorphism on the postprandial TAG response, and should be considered in the design of future studies.

The impact of genotype on the incremental change in TAG response to sequential meal ingestion showed a non-significant trend in the normal weight participants, which is interesting given the tendency for higher overall fasting TAG and postprandial TAG responses in the overweight/obese group. Calculation of TAG IAUC represents both the production and clearance of TAG-rich lipoproteins (TRLs) from the circulation, two highly insulin dependent processes also known to be modulated by TNF-α. High levels of TNF-α have been shown to inhibit lipoprotein lipase activity *in vitro* [30] and enhance the production of liver-derived TRL [31] and adiposity has been reported to potentiate the effects of the *TNFA* polymorphism on lipid metabolism and insulin resistance. Defective insulin secretion and action can lead to an exaggerated TAG response to a high fat meal, but we only found a tendency for a higher HOMA-IR in the overweight/obese group, with a lack of an effect of the polymorphism on postprandial NEFA and glucose responses. It is therefore possible that higher background circulating levels of TNF-α observed in obese individuals [32] may have contributed to the higher fasting and postprandial TAG response, and masked the subtle effects of this polymorphism on TRL metabolism. However, TNF- α was not measured in the UK postprandial cohort which makes it difficult to determine the mechanisms underlying the difference in response between the two genotype groups. Although the MECHE data showed a tendency for higher TNF- α in the GG group, this finding was not significant.

A few studies have examined the association between *TNFA* -308G → A polymorphism and postprandial lipaemia but findings have been inconsistent. In obese families, Wybranska et al., found men carrying the A allele to have higher fasting glucose and postprandial TAG and NEFA responses following an oral fat load whilst women had higher HOMA-IR [10]. However, a lack of an association between the polymorphism and postprandial lipid responses have been reported after sequential high fat meals in obese type 2 diabetic individuals [6] and a single meal containing 65.5 g of dairy fat in offspring of patients diagnosed with cardiovascular disease (CVD) [12]. Our findings are in accordance with a study in CVD patients classified with the metabolic syndrome (MetS), where GG homozygotes had a significantly higher postprandial TAG response than A-allele carriers after a single high fat meal (0.7 g fat/kg body weight containing on average 62 g of fat) [11]. Unlike the study of Gomez-Delgado et al. [11], we also observed a significant effect of genotype on fasting glucose, with higher levels in the GG than GA and AA groups combined. Similar observations have been reported in the LIPGENE cohort, with an increased risk of developing fasting hyperglycaemia and MetS in GG homozygotes [33]. Disagreement in the relationship between -308A and -308G alleles and fasting glucose and lipid responses may reflect the study populations (type 2 diabetic, obese, MetS and familial history of CVD) since expression of the biochemical phenotype of this polymorphism has been proposed to be dependent on the presence of other CVD risk factors [6]. This may explain the similarity of our findings with the Spanish study since 53 % of men in our cohort could be classified with the MetS [16]. In addition, the differences in the allelic effects could be due to the variations in the genotype frequencies across the European cohorts as shown by dbSNP (http://www.ncbi.nlm.nih.gov/projects/SNP/snp_ref.cgi?rs=1800629). Hence, it is possible that the same allelic effect seen in the Spanish study [11] but not seen in the Polish study [10] could be due to differences in the linkage disequilibrium map across European populations.

A limitation of our study is the inability to replicate our findings in a suitable large postprandial cohort. Although we used the Irish postprandial study (MECHE) [15] as a replication cohort, we were unable to replicate the findings due to several reasons. Whilst the two populations were examined for impact of *TNFA* -308G → A polymorphism on postprandial lipaemia, there may have been other genetic variations [34] which could have an impact, but were not controlled for in this analysis. Furthermore, the replication cohort underwent an OLTT which did not follow the same post-prandial meal composition as that of the UK postprandial cohort and hence could have had different consequences on the parameters considered. Also, we cannot rule out the fact that our findings in the UK postprandial cohort could be a chance finding due to small sample size (*n* = 67). However, this is unlikely given the previous link between the *TNFA* -308G → A polymorphism and BMI [35, 36], fasting glucose [37] and postprandial lipaemia [10, 11]. Another limitation is the difference in the proportion of men and women in each of the genotype groups within the BMI sub-groups but to account for this the general linear model was adjusted for gender and age in all of the analyses. Also, the gender- specific association of the SNP with postprandial TAG in our cohort confirms our previous findings in men [17].

## Conclusions

Our findings indicate that the *TNFA* -308G → A polymorphism is independently associated with BMI, fasting glucose concentration and incremental postprandial TAG response to sequential meal ingestion. Further studies are warranted to investigate the mechanisms underlying the effect of the *TNFA* polymorphism on glucose and TAG metabolism, and determine whether BMI is an important variable which should be considered in the design of future studies.

## Abbreviations

AUC: Area under the curve
BMI: Body mass index
IAUC: Incremental area under the curve
NEFA: Non-esterified fatty acid
TAG: Triacylglycerol
TNFA: Tumor necrosis factor – alpha
